# Digital job demands and healthcare workers' workplace well-being: the mediating role of job and personal resources

**DOI:** 10.3389/frhs.2026.1743364

**Published:** 2026-02-27

**Authors:** Parisa Afshin, Barbara Rebecca Mutonyi, Erlend Nybakk

**Affiliations:** School of Economics, Innovation, and Technology, Kristiania University of Applied Sciences, Oslo, Norway

**Keywords:** digital innovations, digital job demands, employee well-being, healthcare workers, resources

## Abstract

**Background:**

Digital innovations are constantly reshaping health care, affecting health care workers’ practices and well-being both positively and negatively. To balance this dual impact, it is essential to understand the specific demands introduced by digital innovations and to assess whether existing personal and organizational resources are related to these demands. Subsequently, this study aims to examine the extent to which resources, such as digital innovation support, autonomy, and resilience, mediate the relationships between digital job demands and job satisfaction and thriving at work.

**Methods:**

We conducted a cross-sectional survey of 292 healthcare workers in the UK recruited via Prolific using nonprobability purposive sampling based on predefined eligibility criteria. The covariance-based structural equation modeling was performed in R version 4.4.3 to test hypothesized relationships and mediations.

**Results:**

Digital technology support, autonomy, and resilience were each positively associated with well-being outcomes; for example, digital innovation support was positively related to thriving at work (*β* = 0.169, *p* = 0.016), and resilience was associated with job satisfaction (*β* = 0.232, *p* = 0.003). Digital system overload showed a significant relationship only with thriving at work, while digital work overload showed no direct associations with thriving at work or job satisfaction; corresponding direct-effect hypotheses were therefore not supported at the 5% level. Two mediations were supported: digital innovation support showed a significant indirect effect between digital system overload and job satisfaction, and resilience showed a complementary mediating effect between digital system overload and thriving at work.

**Discussion:**

In highly digitalized settings, digital job demands did not consistently operate as direct stressors. In healthcare context, resources including autonomy, digital innovation support and resilience were positively related to well-being and, in some cases, operated as mediating mechanisms linking digital system overload to well-being outcomes. This study extends the job demands-resources model by highlighting the importance of mediating role of resources in highly digitalized healthcare work.

## 1 Introduction

In the aftermath of the COVID-19 pandemic, healthcare systems across countries have experienced a rapid and often uneven intensification of digital innovation (DIs) adoption, reshaping work practices and professional roles for healthcare professionals (1). In this paper, DIs in health care is defined as the creation or adoption and exploitation of novel technologies, processes, or systems, such as electronic health records (EHRs), telemedicine, and AI-driven diagnostics, that leverage digital tools and data to improve health care delivery, patient outcomes, and operational efficiency (2). Empirical evidence suggests that while DIs were essential for maintaining service delivery and healthcare resilience, they also exposed gaps in organizational support, training, and workload management for healthcare workers (1, 3–6). These developments underscore the urgency of examining not only whether digital innovations are adopted, but how they shape everyday work conditions and employee well-being in already strained healthcare environments.

This is a critical issue, as accumulating empirical evidence indicates that adopting DIs has a dual impact on healthcare workers' well-being. On the one hand, the adoption of DIs in healthcare has been associated with positive employee outcomes, including greater resilience during periods of environmental uncertainty, such as the COVID-19 pandemic, increased engagement, and reduced burnout (7–10). For instance, empirical studies show that adopting artificial intelligence reduces burnout among radiologists (11). On the other hand, DIs have introduced new and intensified work demands, including increased or duplicated workloads, technological frustration, digitally induced stress, and limited opportunities for healthcare workers to adequately learn and adapt to new systems (12, 13). This dual impact underscores the need to further our understanding of how digital innovations affect healthcare workers' well-being, particularly in a sector already characterized by high levels of burnout and turnover (14, 15).

However, previous studies on the impact of digitalization in the health care sector have frequently focused on patient outcomes (5, 16), organizational outcomes (17, 18), and adoption behavior, incentive mechanisms, and digital competence of the health care workers (19–22) and have often neglected to adequately address the healthcare workers' well-being. Understanding digital innovations and their impact on health care workers is crucial, as their well-being not only influences individual outcomes but also predicts organizational outcomes, such as absenteeism and voluntary turnover (15, 23, 24), and has a significant effect on patient care quality and outcomes (14, 25–28). This is further argued by Xu et al. (29) in their recent systematic meta-analysis that explored the gaps pertaining to the current understanding of the role of digitalization on employees well-being. This highlights a pressing need to better understand how DIs and evolving workplace conditions affect healthcare workers' well-being, especially given that job characteristics can have a profound impact on employee well-being (30). This gap motivates the main objective of the current study: To what extent demanding digital job characteristics impact employee well-being and through which job and personal resources these effects occur.

To address the above objective, we adopt the Job Demands-Resources (JD-R) model (31, 32). Recent applications of the JD-R model have increasingly emphasized its relevance for understanding well-being in complex, technology-intensive work environments, highlighting the importance of context-specific demands and resources (33–35).

The JD-R model proposes that the health and well-being of the employee results from the balance between available resources and job demands (30). Job demands refer to aspects of work that require sustained physical, emotional, or cognitive effort and may impair well-being by depleting energy, whereas job resources are physical, psychological, social, or organizational factors that support goal attainment, reduce the costs of demands, and foster personal growth and development (31, 36). In increasingly digitalized work environments, digital technologies may function both as resources that enhance efficiency and autonomy, and as sources of new job demands, such as digital overload, constant connectivity, and system complexity, which can undermine well-being and performance (37). These digitally induced demands align closely with technostress theory, which highlights how technology use may generate strain when employees feel overwhelmed, insecure, or unable to adapt to digital systems (38, 39). In this study, we examine the demands posed by DIs. Notably, the JD-R model emphasizes that job resources play a critical role in shaping employees’ responses to demanding work conditions. Resources may explain how job demands translate into employee well-being outcomes and, under certain conditions, contribute to positive well-being outcomes by supporting motivation, resilience, and engagement (31, 40). In the context of digital innovation, prior research suggests that organizational and psychological resources are particularly salient for shaping how employees experience and respond to digitally transformed work environments (41).

**Figure 1:**
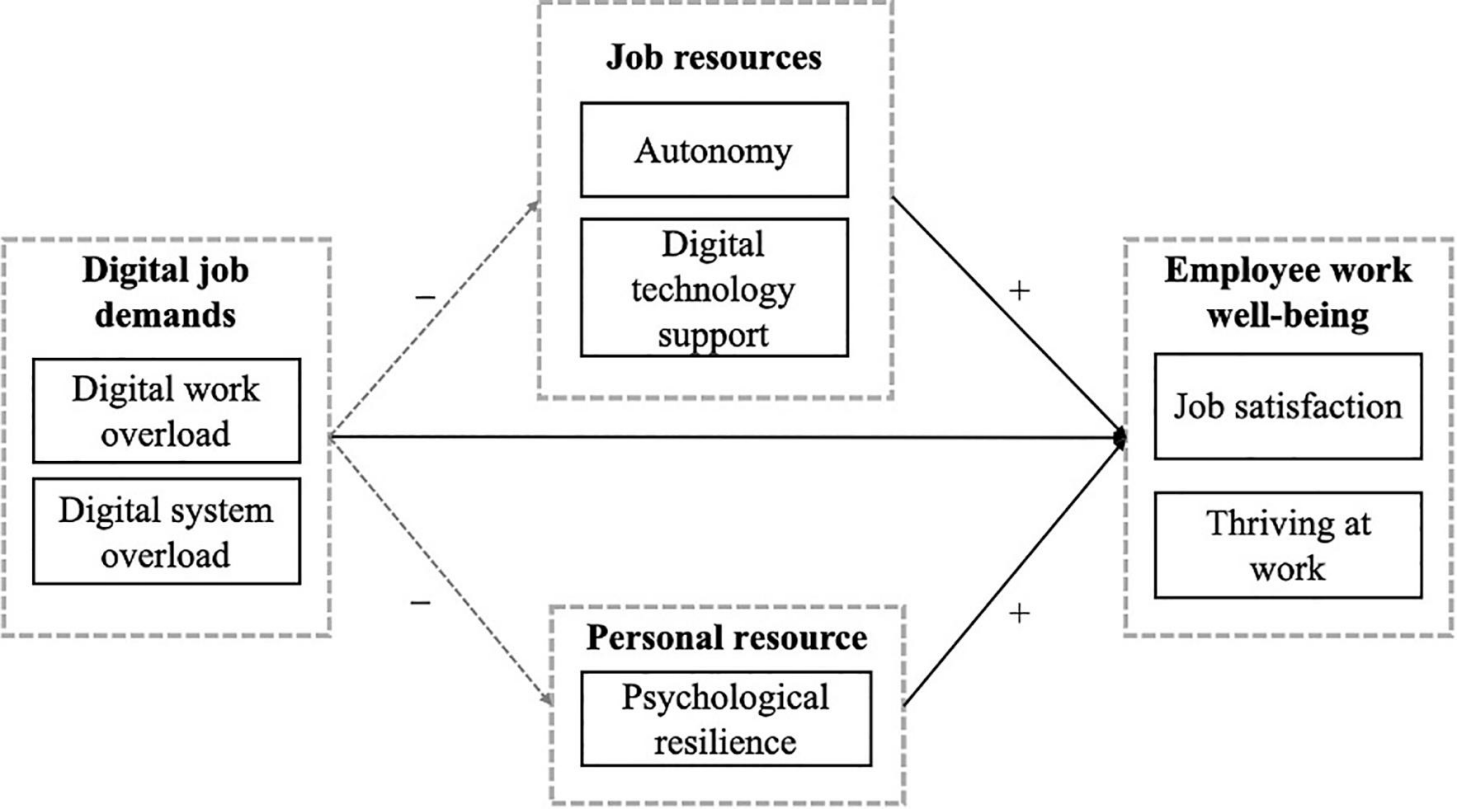
Conceptual model of digital demands, resources, and well-being among health care workers.

The logic on the role of resources is grounded in conservation of resources theory (COR), which emphasizes the salience of resources when individuals face demanding work environments (30, 36, 42). Nonetheless, the JD-R model is flexible and does not propose a fixed set of job characteristics as universally important for employee well-being. Instead, the JD-R model emphasizes the uniqueness of organizational and occupational contexts, which may be characterized by different job demands and resources (36). In this study, we adopt the revised JD-R model proposed and extended by Taris and Schaufeli (43). Specifically, the revised and extended JD-R model posits resources as mediators on the relationship between job demands and work outcomes. The extended JD-R model highlights that job resources have fundamentally mediational qualities (43), and empirical evidence from recent studies of Fleischer & Wanckel (44) supports this claim. However, there exist two major theoretical and empirical gaps: 1) the role of both personal and organizational resources in shaping responses to digital job demands and maintaining well-being has received limited attention. 2) Many of the existing research on digital innovations and employee well-being has primarily focused on negative outcomes, such as burnout and technology induced stress (38, 45), leaving gaps in understanding how these negative effects can be mitigated and to what extent resources may foster positive well-being outcomes (46). Thus, building on the dual-process view of the JD-R model, the current study explores the extent to which resources, such as digital technology support (DTS), autonomy, and resilience, may mediate the negative effects of digital job demands on job satisfaction and thriving at work. The conceptual model guiding this study is presented in Figure 1.

By addressing the above gap, this study makes four key contributions to the literature and practice on digitalization and employee well-being in healthcare. First, it addresses the limited attention paid to healthcare workers' well-being in the context of digital innovations, moving beyond the predominant focus on patient and organizational outcomes. Second, it extends the JD-R model by empirically examining how digital job demands relate to well-being through psychological and organizational resources in highly digitalized healthcare settings. Third, it reassesses the relevance of commonly emphasized resources, such as autonomy and resilience, as mediating mechanisms, offering new insights into their role in contemporary digital work environments. Finally, the study provides practical implications for healthcare organizations by identifying resources that may through which digital job demands are associated with employee well-being and support employee well-being.

### 1.1 Conceptual model

As illustrated in Figure 1, the conceptual model of this study includes both direct and indirect relationships. Following the JD-R model, this study proposes that digital job demands (DJDs), consisting of digital system overload (DSO) and digital work overload (DWO), are directly and negatively related to job satisfaction (JS) and thriving at work (TAW). Based on JD-R model and the importance of resources (30, 36), Figure 1 propose that resources such as autonomy, resilience and digital technology support (DTS), are positively and directly related to JS and TAW. In line with previous empirical findings arguing for resources as mediators (44, 47), this study proposes resources consisting of DTS, autonomy and resilience as mediators in intervening the negative impact of job demands (30, 36) on JS and TAW. As such, as shown in Figure 1, this study proposes that the role of job demands on employees' well-being can be mediated by the three proposed resources (44, 47). Note that in Figure 1, the straight lines indicate direct relationships, while the dotted line indicates indirect relationships. The proposed hypotheses are illustrated in Figure 1 and are further discussed below.

## 2 Review of the literature and hypothesis development

### 2.1 Employee well-being

General theories of individual well-being are often derived from two major viewpoints. The first is the hedonic view, which emphasizes the pursuit of pleasure and subjective happiness, while minimizing pain as an essential component of well-being (48). The second is the eudemonic view, which emphasizes finding meaning, purpose, and self-realization as central to well-being (48). Although some studies of well-being have focused on the hedonic perspective while others have focused on the eudemonic perspective, the findings of these studies have revealed that individuals who pursue both hedonic and eudemonic well-being often experience greater overall well-being and live a “full life” (49, 50). This notion suggests the importance of including both perspectives in understanding employee well-being. Simultaneously, well-being is a complex and multifaceted construct that extends beyond ill-health avoidance to positive states (51).

Drawing on the Conservation of Resources perspective (52) and Job Demands–Resources framework (31) and hedonic and eudaimonic perspectives on well-being (53), in this study, employee well-being is conceptualized as an outcome that emerges from employees' ability to maintain, recover and build psychological, physical, and social resources in response to demanding work conditions and environment, and manifest through both hedonic (i.e., pleasure and satisfaction) and eudaimonic (i.e., vitality, learning, and personal growth) dimensions. This conceptualization is applicable to workplace well-being, and it is aligned with the JD-R model, which emphasizes the balance between resources and demands as critical to employee well-being and motivation. Accordingly, the present study adopts two notions, “thriving at work” and “job satisfaction”, as indicators of employees' workplace well-being.

First, job satisfaction (JS) reflects the hedonic aspect of workplace well-being, emphasizing pleasure, contentment, and satisfaction with work (54). JS suggests the happiness and fulfillment that individuals derive from their work, and it has previously been found to be closely related to organizational outcomes, such as productivity and retention, as well as individuals' overall well-being (55–59). Recent evidence from healthcare contexts further provides additional empirical support for the mediating role of JS in linking personal and leadership-related resources to employee performance and well-being (60). The increasing integration of DIs in the workplace necessitates an agile approach to examining their impact on JS, as DIs often introduce job demands that can negatively influence satisfaction (61–63). For instance, research examining physician JS has shown that electronic health records can diminish professional satisfaction by creating time-intensive tasks and disrupting patient care (26). Nonetheless, a better understanding of the role of digital job demands in JS for a broader sample that includes health workers who fall outside the physician category is lacking.

Second, thriving at work (TAW) reflects a more eudaimonic view of workplace well-being. TAW is defined as a psychological state characterized by the joint experience of vitality (energy and enthusiasm) and learning (growth and development) (64, 65). This dual construct captures both the affective and developmental components of well-being, integrating hedonic and eudaimonic elements (66, 67). Empirical studies have linked TAW to a variety of positive outcomes, including improved mental and physical health, increased life satisfaction, improved job performance, and stronger work engagement (68–70). For example, Walumbwa et al. (68) highlighted how TAW supports sustainable organizational performance through its multilevel effects on individuals and teams.

Despite its growing relevance, TAW remains underexplored in the context of digital transformation in health care. While digital innovations are often implemented to improve system efficiency and organizational sustainability, their effects on employee development and vitality are not well understood. This study addresses this gap by examining how digital job demands are related to TAW to inform strategies that support the well-being of health care workers and, by extension, their sustainable performance. In doing so, and in line with Walumbwa et al. (68), Williams et al. (71), and Zhai et al. (69), this study provides further insight into how organizations, specifically in health sector, can enhance work performance through the well-being of health care workers.

### 2.2 Digital job demands

While digital innovations (DIs) can offer resource-enhancing benefits, such as automating routine tasks, they may also introduce additional job demands by increasing workload and complexity (72). In this study, we focused on demands caused by DIs. We adopt Scholze and Hecker's (73) definition of digital job demands (DJDs) as job demands that arise from the use of DIs, including information overload, system inefficiencies, and new skill requirements. Prior research shows that DJDs can undermine JS and organizational commitment (12), blur work-life boundaries (74), and increase turnover intentions and workplace detachment (75). A recent meta-analysis further revealed that digital stressors, such as technostress, are associated with increased exhaustion and lower employee well-being (76). Yet, despite the growing body of research, empirical evidence from the health care sector remains limited (12, 17). This gap is critical, as the JD-R model emphasizes that job demands are context-dependent and may vary across sectors and organizations (36).

Moreover, most existing studies focus on the effects of individual technologies, such as mobile devices (77), rather than on the broader interplay of multiple systems. To address this, the present study adopts a cumulative view of DJDs, reflecting the various digital systems and tools health care workers interact with daily. This approach aligns with calls for a more holistic understanding of how digital innovations influence employee well-being (46). Specifically, we examine two central types of DJDs that are particularly relevant in the health care sector: digital system overload (DSO) and digital work overload (DWO).

Previous research has shown major challenges related to digital systems in health care (1, 78), and work overload is also a common issue in health sector (79). DSO reflects the complexity and feature excess of digital tools, such as electronic health records (EHRs), which can overwhelm users and reduce usability (72). Notably, the time-intensive navigation of EHRs disrupts patient care, which also results in decreased professional satisfaction (26). Thus, DWO occurs when digital tools that are intended to save labor increase tasks, volume, and pace (72). Previous studies have shown that the frequent use of digital technologies, while improving efficiency, often increases perceived workload (80) and decreases vitality (81). In this study, we propose that DJDs, as represented by DWO and DSO, are negatively associated with employee well-being, as specified as JS and TAW. The suggested hypotheses are formulated as follows (see Figure 1):

***H1*** *: DWO (a) and DSO (b) are negatively associated with JS.*

***H2*** *: DWO (a) and DSO (b) are negatively associated with TAW.*

### 2.3 Organizational and personal resources

Nielsen et al. (82) categorize workplace resources into four types: i) personal-level (e.g., self-efficacy and competence), ii) group-level (e.g., social support and teamwork), iii) leader-level (e.g., leadership style and leader-member exchange), and iv) organizational-level (e.g., job design and management practices) resources. These resources can have both direct and indirect relational effects on employee well-being outcomes in tackling various work demands (36) on employee well-being. Therefore, resources can enhance well-being by enabling effective performance and coping (82). For instance, previous research shows that work ability as a personal resource can mediate the relationship between job demands and emotional exhaustion among older Italian teachers (83). However, to the best of the authors' knowledge, few studies in health services research have explored the role of resources on health care worker's well-being, here found in JS and TAW. Consequently, as depicted in the study's conceptual model (Figure 1), this paper focuses on two levels of resources; i), organizational-level resources that is represented by digital technology support (DTS) and autonomy, and ii), and the personal-level resource represented by psychological resilience.

Consistent with prior research highlighting resource-based mechanisms of empowerment in healthcare (84), this study conceptualizes digital innovation support and autonomy as contextual resources that can enable employees to mobilize their capacities and adapt to digital demands. In detail, digital technology support (DTS) includes technical assistance for digital tools, while autonomy, reflects on employees' control over task execution. Prior studies suggest that DTS and autonomy can aid employees in managing DJDs and achieve work goals (82, 85) by enhancing technological coping (86, 87) and fostering control (23), respectively. At the personal level, psychological resilience is defined as the capacity to adapt to stressors. Resilience has been shown to facilitate coping and support positive adaptation, thus promoting employee well-being (42, 85). In line with the motivational pathway of the JD-R model, these resources are expected to positively contribute to well-being outcomes such as JS and TAW. The following hypotheses are therefore proposed:

***H3*** *: DTS (a), autonomy (b), and psychological resilience (c) are positively associated with JS.*

***H4*** *: DTS (a), autonomy (b), and psychological resilience (c) are positively associated with TAW.*

### 2.4 Mediating role of digital technology support (DTS)

Within the JD-R framework, digital technology support (DTS) represents an organizational-level job resource that directly targets the demands introduced by digital systems. DTS encompasses technical assistance, training, and organizational support provided to employees for the effective use of digital tools. For example, IT professionals help in navigating electronic health records (EHRs) or resolving system-related issues (30). Although digital innovations aim to streamline tasks and improve efficiency, their implementation often introduces new complexities, increases cognitive load, and disrupts workflows, especially when adequate support structures are absent (64). For instance, Alwali and Alwali (88) found that digital technologies yield the most positive outcomes when they are accompanied by effective and emotionally intelligent leadership that promotes engagement and psychological safety. In such cases, digital job demands may impair employees' JS and sense of vitality unless they are counterbalanced by tailored support mechanisms (87).

In digitally intensive health care environments, DTS becomes particularly crucial. It enables employees to manage system overload and the digital workload by boosting their confidence, alleviating frustration, and supporting smooth task execution. A supportive climate that includes training, access to troubleshooting, and ongoing technical assistance has been shown to enhance employee engagement with technology and strengthen self-efficacy (87, 89, 90). These support structures help minimize workflow interruptions, reduce stress related to system use, and enable a focus on core care responsibilities—ultimately supporting JS and TAW (87, 91, 92). Although the positive effects of organizational support are well established, the specific mediating role of DTS in shaping the relationship between digital job demands and employee well-being remains underexplored, particularly in health care settings (46). Therefore, this study contributes fresh insights into DTS as a mediator for the relationship between DJDs and well-being outcomes: JS and TAW. Based on this rationale, the following hypotheses are proposed:

***H5a*** *: DTS mediates the relationship between DWO and JS.*

***H5b*** *: DTS mediates the relationship between DWO and TAW.*

***H5c*** *: DTS mediates the relationship between DSO and JS.*

***H5d*** *: DTS mediates the relationship between DSO and TAW.*

### 2.5 Mediating role of autonomy

Autonomy is understood as one's ability to self-govern one's work and includes work scheduling, decisions, and methods (93). Since job strain is particularly caused by the combination of high job demands and low job control (30), autonomy is considered a cornerstone resource within the JD-R model (30, 31). Digital job demands, such as complex systems or rigid workflows, can severely limit employees' sense of control. Therefore, autonomy becomes a meaningful mechanism to preserve well-being under such conditions. This aligns with prior JD-R research showing that strain is often amplified when high demands are combined with low job control (36). Autonomy satisfies one of the core psychological needs (94), increasing JS and TAW (61).

Autonomy-supportive environments improve engagement and psychological health, whereas controlling environments diminish vitality. In the digital context of health care, autonomy may empower workers to manage DJDs, such as EHR complexity, offering flexibility, preserving satisfaction, and learning (95). For instance, studies shows that nurses with scheduling control report greater TAW amidst technological pressures (82). Similarly, a study by Zhang et al. (41) on Chinese health care personnel revealed that perceived job resources, such as autonomy and support, are positively associated with TAW (61). Another study indicated that support from colleagues and autonomy can function as key mediators in the relationship between job demands and employees' social well-being outcomes, such as connectedness in the workplace (47). Additionally, the JD-R model is often enriched through integration with complementary theories such as the conservation of resources (COR) theory.

COR suggests that individuals strive to protect, retain, and build valuable resources, particularly under conditions of threat or loss (42). From this perspective, autonomy may serve as a transmission mechanism through which employees preserve well-being by offsetting the resource depletion caused by digital job demands. An indication that autonomy serves as mediator between digital job demands and well-being. Recent research by Fleischer and Wanchel (44) further demonstrates that autonomy can mediate the relationship between digital overload and JS, supporting the view that autonomy functions as a mechanism through which digital demands influence well-being. When autonomy is supported, employees are more likely to adapt flexibly, preserve motivation, and sustain JS and TAW. However, its mediating role in mitigating the effects of DJDs on positive well-being remains underexplored in health services research (13). Thus, the following hypotheses are formulated:

***H6a*** *: Autonomy mediates the relationship between DWO and JS.*

***H6b*** *: Autonomy mediates the relationship between DWO and TAW.*

***H6c*** *: Autonomy mediates the relationship between DSO and JS.*

***H6d*** *: Autonomy mediates the relationship between DSO and TAW.*

### 2.6 Mediating role of psychological resilience

Resilience is a positive psychological capacity that enables individuals to recover from setbacks, adapt to stress, and maintain functioning under pressure (96). As a personal resource within the extended JD-R model, resilience plays a critical role in how individuals perceive and respond to job demands. Previous studies that have explored the resilience of employees have shown that psychological resilience is a crucial positive resource for overcoming stressors and challenges at work (97, 98). The reason is that psychological resilience reframes challenges as growth opportunities (99). In health care, resilient workers thrive amidst job stressors (100, 101) and report higher levels of JS and performance (99).

Previous research has revealed that DJDs, such as system complexity or task overload, are moderated by psychological resilience through sustained energy and learning, which is achieved by buffering their disruptive effects (102). Despite its relevance, studies exploring psychological resilience as a mediating factor in digital health care contexts are limited (23, 103). This research gap is particularly evident in studies focusing on discussing psychological resilience in terms of fostering positive outcomes rather than merely reducing strain (57). A meta-analysis indicated that personal resources received less attention from research on facing demands than did organizational resources (82). As a personal resource for coping with the negative impacts of digitalization, psychological resilience has received less attention than other individual factors, such as personality traits or self-efficacy (46). This oversight is especially notable in digital health care contexts, where rapid technological changes may challenge workers' emotional and cognitive resources. A recent study by Bayram Değer et al. (104) explored the mediating role of psychological resilience among Turkish health care workers and found empirical support that psychological resilience mediated the relationship between workplace violence and job stress. Though the recent study of Bayram Değer et al. (104) focused on workplace violence and job stress, it argued for variations in factors that might be attributional for psychological resilience as a mediating factor. In line, this study offers an extension of knowledge in the current gap in the literature by empirically exploring the mediating role of psychological resilience on DJD and employee well-being in the health care sector.

Importantly, the flexibility of the JD-R model allows researchers to explore a range of roles for personal resources beyond their traditional use as moderators. As Schaufeli and Taris (105) state: “Personal resources can be integrated into the JD-R model in various ways… they can be integrated as mediators, moderators, ‘third variables,’ antecedents of job demands and job resources, or as any combination of these.” This theoretical openness and invitation, combined with the underexplored role of resilience in digitalized healthcare settings, provides a strong foundation for investigating resilience as a mediating mechanism—a process through which digital job demands may impact employee well-being outcome such as JS and TAW. We therefore propose the following hypotheses:

***H7a*** *: Psychological resilience mediates the relationship between DWO and JS.*

***H7b*** *: Psychological resilience mediates the relationship between DWO and TAW.*

***H7c*** *: Psychological resilience mediates the relationship between DSO and JS.*

***H7d*** *: Psychological resilience mediates the relationship between DSO and TAW.*

## 3 Methodology

The aim of this study is to advance the understanding of the extent to which DJDs affect the well-being of health care employees and the extent to which key psychological and organizational resources influence and mediates these relationships within the JD-R framework. As illustrated in the conceptual model of this study (Figure 1), the analysis focuses on DWO and DSO as central DJDs, on DTS, autonomy, and psychological resilience as central resources, and on JS and TAW as central employee well-being factors.

To address these aims, data were collected through a survey questionnaire administered to health care employees. The data were analyzed via covariance-based structural equation modeling (CB-SEM) in R software to test the hypothesized relationships among DJDs, resources, and well-being outcomes.

### 3.1 Sample procedure and data collection

Following the emphasis of the JD-R model on context-specific assessments of demands and resources (36), we initiated a review with ten health care professionals (nurses and doctors from various hospital units). First, we discussed what are the common DJDs that they face at work. Then, based on their feedback, we created and presented a list of constructs using established measurements. They were asked to assess the face validity of the constructs, particularly those related to digital job demands (DJDs) and psychological and organizational resources. Drawing on the literature on technostress and technology overload (72, 91), an initial pool of constructs with established measurements, including techno-invasion, information overload, work overload, and system overload was presented. Based on their professional experience, the experts identified system overload, work overload, and information overload as the most relevant DJDs for their daily work. Then the adapted items from the established measurements were slightly refined using expert feedback.

Next, prior to conducting the main studies, we initiated a pilot study (*n* = 60) to ensure the quality of questionnaire and to test our model. The pilot was conducted via the Prolific online survey platform. The participants provided structured feedback on the clarity and relevance of the items. This pilot phase also allowed us to test measurement quality via confirmatory factor analysis (CFA), partial correlations, and simple regressions to assess the face validity of the proposed hypotheses, as shown in Figure 1. Based on the pilot results, minor modifications were made to improve item clarity and model structure.

The main study was then conducted in December 2024, also via Prolific. We recruited 500 UK-based health care workers using nonprobability purposive sampling based on predefined eligibility criteria. Prolific is a well-established platform that has been used in numerous peer-reviewed studies within the field of business research [e.g., Ellis et al., (106)], and the quality of the data obtained through the platform is generally considered to be high (107). In line with Douglas et al. (107), online platforms such as Prolific tend to yield high response rates owing to a combination of participant incentives, prescreening tools, and built-in quality controls. Using Prolific offers several benefits, including decreased time used in the recruitment of participants, access to a larger population based on preselected criteria, shorter response times in the responses collected, the recruitment of high-quality samples, almost instant comparable data with a decreased number of missing observations and complete anonymity for the respondents (107–110). Consistent with the recommendations of Aguinis et al. (111) and Douglas et al. (107), we employed Prolific's prescreening tools to target active health care professionals and to ensure sample rigor. The criteria included current employment in a clinical role (e.g., doctors, nurses, paramedics, emergency dispatchers, or medical services personnel), residency in the UK, full proficiency in English, and the exclusion of respondents in temporary, intern, or apprenticeship roles.

The total number of eligible participants was 500, of which a total of 300 responses were collected within the time limit (response rate = 60%). Nevertheless, of the 300 responses retained, 8 samples were excluded because of noncompliance with the time-based quality metric, leaving a final sample of 292 valid responses. No missing data were observed in the final dataset. The sample met the recommended minimum for covariance-based structural equation modeling (CB-SEM), which requires approximately *N* = 200 (86).

The participants included nurses, doctors, paramedics, emergency dispatchers, and other health care service personnel. The sample was predominantly female (77%), with most participants working as nurses (65%), holding a bachelor's degree (55%), and working full-time (65%). Leadership roles were also common (35% team leaders). Descriptive statistics are detailed in Supplementary Appendix A. All participants were fully anonymous, participation was voluntary, and informed consent was given by proceeding beyond the initial survey introduction screen.

### 3.2 Ethical considerations

Data collection was conducted in accordance with the ethical guidelines of the Norwegian Agency for Shared Services in Education and Research (SIKT) and the European General Data Protection Regulation (https://gdpr.eu/what-is-gdpr/). As no identifiable personal data, IP addresses, or clinical interventions were involved, the study was approved by SIKT without assignment of a reference number. Anonymity was ensured by using a secure survey platform, Nettskjema (http://www.nettskjema.no), that automatically remove identifying information. Data are stored securely at Kristiania University of Applied Sciences. Participation was voluntary, and informed consent was obtained electronically prior to survey completion.

### 3.3 Measurement instruments

The study survey adapted items from established measures used in previous studies (see Table 1). All constructs were measured via a 7-point Likert scale (1 = strongly disagree, 7 = strongly agree), asking respondents to answer while reflecting on the digital technologies that they are using the most in their daily health care work, such as electronic health records (EHRs), telemedicine platforms, and clinical decision support systems (CDSSs). Table 1 details the constructs, subconstructs, items' code, adapted items, and sources. Autonomy was measured using five items from Morgeson and Humphrey (93), resilience with six items from Smith et al. (112), DTS with five items adapted from Day et al. (87), TAW with eight items capturing learning and vitality from Porath et al. (66), JS with four items from Thompson and Phua (59), DSO with four items from Wisniewski and Lu (72), and DWO with three items from Carlson et al. (75).

**Table 1:** Measurement instruments details.

Construct	Sub-construct	Item code	Adapted items	Sources
Autonomy		aut1	The job allows me to make my own decisions about how to schedule my work.	Morgeson & Humphrey (93)
		aut2	The job allows me to plan how I do my work.	
		aut3	The job gives me a chance to use my personal initiative or judgment in carrying out the work.	
		aut4	The job allows me to make a lot of decisions on my own.	
		aut5	The job provides me with significant autonomy in making decisions.	
Resilience		res1	I tend to bounce back quickly after hard times	Smith et al., (96)
		res2	I have a hard time making it through stressful events (R)	
		res3	It does not take me long to recover from a stressful event	
		res4	It is hard for me to snap back when something bad happens (R)	
		res5	I usually come through difficult times with little trouble	
		res6	I tend to take a long time to get over setbacks in my life (R)	
Digital technology support (DTS)		icts1	The technology support team is available at work when I need it.	Day et al., (87)
		icts2	Our information technology support team are helpful.	
		icts3	My organization's technology support team respond promptly to my technical challenges.	
		icts4	My technology department teaches me to solve problems.	
		icts5	My organization's technology support team updates me on issues and solutions.	
Thriving at work (TAW)	Learning	TAW1	I find myself learning often.	
		TAW2	I continue to learn more as time goes by.	Porath et al., (66)
		TAW3	I see myself continually improving.	
		TAW4	I am not learning (R).	
	Vitality	TAW5	I feel alive and vital.	
		TAW6	I have energy and spirit.	
		TAW7	I do not feel very energetic (R).	
		TAW8	I feel alert and awake.	
Job satisfaction (JS)		job1	I am looking forward to each new day.	Thompson & Phua (59)
		job2	I find real enjoyment in my job.	
		job3	I like my job better than average person.	
		job4	Most days I am enthusiastic about my job.	
Digital job demand (DJD)	Digital system overload (DSO)	demsys1	I am often distracted by unnecessary features in the digital technologies I use for my job.	Wisniewski & Lu, (72)
		demsys2	Poor user interface design in digital tools often reduces my productivity in daily work.	
		demsys3	The software I use at work often includes unnecessary features, making it harder to use effectively for my main tasks.	
		demsys4	The software applications I use at work are often slow or prone to freezing, which disrupts my productivity.	
	Digital work overload (DWO)	demwork1	My work demands have increased due to digital technologies.	Carlson et al., (75)
		demwork2	The technology at my job requires a lot from me.	
		demwork3	My work tasks have increased due to digital technologies.	

### 3.4 Data analysis

The conceptual model and the hypothesized relationships were tested via CB-SEM in R 4.4.3 software. Analyses were conducted using the following packages in R: psych (113), lavaan (114), semTools (115), and polycor (116). The analysis followed a two-step process.

In the first step, the measurement model was assessed. Although Likert-type items are ordinal, simulation studies have shown that when items have five or more response categories and approximately symmetric thresholds, robust maximum likelihood estimation (MLR) based on the Pearson covariance matrix yields unbiased estimates comparable to those obtained with categorical estimators such as WLSMV (117). Accordingly, the measurement model was estimated using MLR with Huber–White robust standard errors and the Yuan–Bentler scaled *χ*^2^ statistic. Validity and reliability were assessed via factor loadings, cross-loadings, Heterotrait–Monotrait ratio correlations (HTMT), and variance inflation factor (VIF).

In step 2, CB-SEM was used to test the hypothesized relationships (H1–H7) employing robust maximum likelihood (MLR) estimation. First, model fit was evaluated via the comparative fit index (CFI), Tucker‒Lewis index (TLI), root mean square error of approximation (RMSEA), standardized root mean square residual (SRMR), and coefficient of determination (R^2^). Afterward, the direct relationships (H1–H4) and the mediations (H5–H7) were evaluated with 5,000 bootstrap samples in the mediation analysis (118).

The results of mediation were interpreted according to Zhao et al. (119), in which mediation can be categorized into five types based on the significance and direction of the indirect and direct effects: (1) indirect-only mediation, where only the indirect path is significant and the direct path is not; (2) complementary mediation, where both the indirect and direct effects are significant and point in the same direction; (3) competitive mediation, where both paths are significant but in opposite directions; (4) direct-only non-mediation, where only the direct path is significant; and (5) no-effect non-mediation, where neither the direct nor the indirect path is significant. This classification provides a theoretically grounded framework for interpreting the nature of mediation beyond traditional Baron and Kenny steps and was applied in this study to determine the type of mediation present in each case.

## 4 Results

### 4.1 Measurement model results

To assess the psychometric properties of the constructs, a confirmatory factor analysis (CFA) was conducted using MLR. The initial model, including all original items, showed a suboptimal but acceptable fit: CFI = 0.847, TLI = 0.831, RMSEA = 0.086, SRMR = 0.078. To improve model fit and ensure construct validity, five items with the weakest standardized factor loadings were removed (all < 0.70) (120). In detail these are *aut1* (*λ* = 0.48), *aut2* (*λ* = 0.53), *TAW1* (*λ* = 0.55), *TAW2* (*λ* = 0.57), and *TAW4* (*λ* = –0.42). Supplementary Appendix B presents the initial measurement model, listing all items prior to purification and indicating which were retained and which were dropped. The revised model demonstrated improved and acceptable fit: CFI = 0.94 and TLI = 0.94 and RMSEA = 0.055 and SRMR = 0.058, supporting the adequacy of the final measurement model. Table 2 presents the final measurement model, including standardized factor loadings, means, and standard deviations after deleting the mentioned items. Composite reliability (CR), Table 2, reporting coefficient omega as common practice (121), was used to assess internal consistency, as it offers advantages over Cronbach's alpha (122). Convergent validity, indicated in Table 2, was evaluated through standardized loadings and average variance extracted (AVE), with values ≥ 0.50 indicating satisfactory convergent validity (122).

**Table 2:** Final measurement model results.

Items	Factor loading	Mean	SD	CR	AVE
DWO		4.68	1.45	0.89	0.73
demwork1	.86				
demwork2	.77				
demwork3	.91				
DSO		4.55	1.28	0.82	0.53
demsys1	.61				
demsys2	.81				
demsys3	.81				
demsys4	.65				
DTS		4.56	1.28	0.87	0.60
icts1	.84				
icts2	.85				
icts3	.87				
icts4	.67				
icts5	.62				
Resilience		4.25	1.28	0.93	0.72
res1	.78				
res2	.78				
res3	.87				
res4	.88				
res5	.85				
res6	.91				
TAW		4.75	0.75	0.74	0.65
TAW3	.62				
TAW5	.88				
TAW6	.95				
TAW7 (R)	-0.72				
TAW8	.81				
JS		4.75	1.32	0.93	0.76
job1	.85				
job2	.92				
job3	.79				
job4	.94				
Autonomy		5.28	1.21	0.90	0.77
aut3	.73				
aut4	.96				
aut5	.88				

SD, standard deviation; CR, composite reliability; AVE, average variance extracted; DWO, digital work overload; DSO, digital system overload; DTS, digital technology support; Resilience, psychological resilience; TAW, thriving at work; JS, job satisfaction; (R), Reversed item. *n* = 292.

The discriminant validity was assessed following recommendations by Henseler et al. (123) that suggested using the HTMT, which has been shown to be a more sensitive and reliable method than the older measures such as the Fornell-Larcker criterion. While the Fornell–Larcker criterion has traditionally been used, recent research highlights its limitations, particularly in variance-based SEM (123). Table 3 presents the HTMT values, all of which were below the conservative threshold of 0.85, supporting the presence of discriminant validity among the latent constructs (123).

**Table 3:** Heterotrait–monotrait ratio of correlations (hTMT).

Constructs	Autonomy	Resilience	DTS	TAW	JS	DSO	DWO
Autonomy	–						
Resilience	0.036	–					
DTS	0.040	0.191	–				
TAW	0.143	0.295	0.235	–			
JS	0.170	0.259	0.257	0.643	–		
DSO	0.065	0.139	0.260	0.156	0.157	–	
DWO	0.125	0.065	0.217	0.041	0.045	0.411	–

DTS, digital technology support; TAW, thriving at work; JS, job satisfaction; DSO, digital system overload; DWO, digital work overload.

To examine potential multicollinearity among the predictor constructs, variance inflation factor (VIF) values were computed. All VIF values were well below the recommended threshold of 3.3 (122), indicating no multicollinearity concerns. Specifically, the VIFs were as follows: Digital Work Overload (1.71), Digital System Overload (1.94), DTS (1.27), Resilience (1.22), and Autonomy (1.05). These results confirm that the constructs are statistically distinct and that collinearity does not bias the parameter estimates.

### 4.2 Structural equation modeling (SEM)

The mediation model demonstrated acceptable fit to the data: CFI = 0.939, TLI = 0.932, RMSEA = 0.059 [90% CI (0.053, 0.065), *p* for RMSEA ≤.05 = .006], and SRMR = 0.066. While the RMSEA indicated a borderline acceptable fit, the CFI, TLI, and SRMR values supported the overall adequacy of the model. The model accounted for 21.7% of the variance in TAW and 19.4% of the variance in JS, as indicated by the R^2^ values. These results suggest that the included predictors and mediators collectively explain a meaningful proportion of variance in the well-being outcomes.

As presented in Table 4, the standardized path coefficients (*β*) and p-values reveal that DWO was not significantly associated with JS (*β* = 0.090, *p* = .271) or TAW (*β* = 0.104, *p* = .196), providing no support for H1a or H2a. DSO was not associated with JS (*β* = –0.139, *p* = .112), but it was significantly negatively associated with TAW (*β* = –0.186, *p* = .030), providing no support for H1b but supporting H2b at the 5% significance level.

**Table 4:** Structural model of the direct relationships.

Hypothesis	Path	Std. Coef (*β*)	*p* value
H1a	DWO → JS	0.090	0.271
H1b	DSO → JS	−0.139	0.112
H2a	DWO → TAW	0.104	0.196
H2b	DSO → TAW	−0.186	0.030*
H3a	DTS → JS	0.246	0.000*
H3b	Resilience → JS	0.232	0.003*
H3c	Autonomy → JS	0.155	0.012*
H4a	DTS → TAW	0.169	0.016*
H4b	Resilience → TAW	0.315	0.000*
H4c	Autonomy →TAW	0.126	0.045*

DWO, digital work overload; DSO, digital system overload; DTS, digital technology support; JS, job satisfaction; TAW, thriving at work.

* *p* < .05 (statistically significant).

In contrast, digital technology support (DTS) showed a statistically significant positive association with both JS (*β* = 0.246, *p* < .001) and TAW (*β* = 0.169, *p* = .016), supporting H3a and H4a. Similarly, psychological resilience was positively related to JS (*β* = 0.232, *p* = .003) and TAW (*β* = 0.315, *p* < .001), supporting H3b and H4b. Autonomy was also significantly associated with both JS (*β* = 0.155, *p* = .012) and TAW (*β* = 0.126, *p* = .045), supporting H3c and H4c.

Subsequently, mediation effects were assessed using robust maximum likelihood estimation with 5,000 bootstrap samples and bias-corrected and accelerated confidence intervals (BCa CI). Following recommended practice (119, 124), the analysis proceeded in two steps. First, indirect effects were estimated, and second, the statistical significance and type of mediation were evaluated based on the joint pattern of indirect, direct, and total effects. Results are summarized in Table 5.

**Table 5:** Results of mediation analysis using bootstrap estimates.

Hypothesis	Path	Effect	Std. Coef (β)	SE	*p*	95% CI	Mediation interpretation		
H5a	DWO → DTS → JS	Indirect	−0.008	0.02	0.682	[−0.053, 0.029]	No mediation		
H5b	DWO → DTS → TAW	Indirect	−0.003	0.009	0.707	[−0.023, 0.012]	No mediation		
H5c	DSO → DTS → JS	Indirect	−0.112	0.045	**0**.013*	[−0.218, −0.038]	**Indirect-only mediation**		
H5d	DSO → DTS → TAW	Indirect	−0.043	0.023	0.057	[-0.097, −0.008]	No mediation		
H6a	DWO → Autonomy → JS	Indirect	0.018	0.014	0.206	[−0.005, 0.049]	No mediation		
H6b	DWO → Autonomy → TAW	Indirect	0.008	0.007	0.271	[−0.002, 0.027]	No mediation		
H6c	DSO → Autonomy → JS	Indirect	0.008	0.021	0.714	[−0.034, 0.053]	No mediation		
H6d	DSO → Autonomy → TAW	Indirect	0.004	0.01	0.726	[−0.017, 0.025]	No mediation		
H7a	DWO → Resilience → JS	Indirect	0.013	0.019	0.477	[−0.021, 0.055]	No mediation		
H7b	DWO → Resilience → TAW	Indirect	0.01	0.014	0.468	[−0.016, 0.039]	No mediation		
H7c	DSO → Resilience → JS	Indirect	−0.073	0.038	0.055	[−0.160, −0.013]	No mediation		
H7d	DSO → Resilience → TAW	Indirect	−0.055	0.026	**0**.033*	[−0.113, −0.011]	**Complementary mediation**		
	DWO → JS	Direct	0.090	0.070	0.281	[−0.055, 0.217]	–		
	DSO → JS	Direct	−0.138	0.120	0.128	[−0.434, 0.042]	–		
	DWO → TAW	Direct	0.105	0.039	0.210	[−0.025, 0.131]	–		
	DSO → TAW	Direct	−0.187	0.066	**0**.036*	[−0.276, −0.019]	–		
	DWO → JS (total)	Total	0.098	0.076	0.199	[−0.045, 0.257]	–		
	DSO → JS (total)	Total	−0.361	0.125	**0**.004*	[−0.627, −0.134]	–		
	DWO → TAW (total)	Total	0.064	0.041	0.114	[−0.013, 0.146]	–		
	DSO → TAW (total)	Total	−0.233	0.07	**0**.001*	[−0.385, −0.109]	–		

DWO, digital work overload; DSO, digital system overload; DTS, digital technology support; JS, job satisfaction; TAW, thriving at work. Mediation was tested with 5,000 bootstrap samples (BCa CI).

* *p* < .05 (statistically significant) highlighted in bold.

The analysis revealed that digital system overload (DSO) exerted two statistically significant indirect effects on employee well-being outcomes. First, DSO had a significant indirect effect on JS through digital technology support (DTS) [*β* = –0.112, SE = 0.045, *p* = .013, 95% CI (–0.218, –0.038)], while the corresponding direct effect was not significant (*β* = –0.138, *p* = .128). This pattern reflects indirect-only mediation, suggesting that DTS fully transmits the negative effect of DSO on JS.

Second, DSO showed a significant indirect effect on TAW via resilience [*β* = –0.055, SE = 0.026, *p* = .033, 95% CI (–0.113, –0.011)]. As the direct effect of DSO on TAW was also significant (*β* = –0.187, SE = 0.066, *p* = .036), this pattern is consistent with complementary mediation, indicating that resilience partially transmits the negative effect of DSO on TAW.

No other mediation paths reached statistical significance. Specifically, the indirect effect of DSO on TAW via DTS (*β* = –0.043, SE = 0.023, *p* = .057) and on JS via resilience (*β* = –0.073, SE = 0.038, *p* = .055) were marginally significant (*p* ≥ .05) and thus do not meet the threshold for mediation. Similarly, none of the indirect paths from digital work overload (DWO) to JS or TAW, through resilience, autonomy, or DTS, were not statistically significant, indicating no mediation. In addition, autonomy did not significantly mediate the relationship between DSO and either outcome.

The pattern of direct and total effects further supports these conclusions. While the direct effect of DSO on TAW remained significant after accounting for mediators (*β* = –0.187, SE = 0.066, *p* = .036), its direct effect on JS was not significant (*β* = –0.138, *p* = .128). All direct effects associated with DWO were non-significant. Notably, DSO had significant negative total effects on both JS [*β* = –0.361, SE = 0.125, *p* = .004, 95% CI (–0.627, –0.134)] and TAW [*β* = –0.233, SE = 0.070, *p* = .001, 95% CI (–0.385, –0.109)]. In contrast, DWO's total effects on both outcomes were non-significant.

In summary, nine hypotheses were supported. The results of our conceptual model show that DTS, resilience, and autonomy were each significantly and positively associated with JS and TAW, supporting hypotheses H3a-c and H4a-c. DWO was not significantly associated with JS or TAW (H1a–H1b not supported). Although DSO was not significantly associated with JS, it showed a significant negative relationship with TAW (H2a not supported; H2b supported).

Regarding mediation relationships (H5a-d, H6a-d and H7a-d), DTS significantly mediated the relationship between DSO and JS (H5c supported), and resilience significantly mediated the relationship between DSO and TAW (H7d supported). The results of all direct and indirect effects are presented in Tables 4, 5. The findings are further discussed in the subsequent section.

## 5 Discussion

This study examined how digital job demands and resources jointly shape healthcare workers' well-being in highly digitalized work environments. Drawing on the Job Demands-Resources (JD-R) model and Conservation of Resources theory, the findings offer new insights into when and how digital job demands affect employee well-being and how organizational and personal resources operate in these relationships. Below, we discuss the theoretical and practical implications of these findings.

### 5.1 Theoretical implications

This study contributes to the literature on digital work and employee well-being by refining how digital job demands and resources operate in highly digitalized healthcare settings. By applying the JD-R model to digitally induced demands and context-sensitive resources, the findings extend existing theory in three important ways.

First, the results challenge the assumption that digital job demands uniformly function as direct stressors. As hypotheses H1a, H1b, and H2a were not supported, we refrain from making strong theoretical claims regarding these hypotheses. However, it is important to reflect that in contrast to dominant technostress perspectives, digital work overload was not significantly associated with job satisfaction or thriving at work, and digital system overload showed no direct association with job satisfaction. Only digital system overload was negatively related to thriving at work. This pattern suggests that digital job demands may not operate uniformly as direct stressors in highly digitalized healthcare settings, diverging from prior research linking technology-related overload to diminished well-being [for example see (12)]. Rather than indicating an absence of strain, these findings can point to the importance of considering how digital demands are appraised and embedded within specific work contexts. Consistent with the challenge–hindrance framework (125), digital demands may function as either hindrances or challenges depending on contextual expectations and available resources. In healthcare, where rapid digitalization is often perceived as necessary and instrumental for care delivery (17), such demands may be more likely to be interpreted as challenges rather than hindrances (10), which may help explain the lack of robust direct negative effects on job satisfaction observed in this study.

Second, the findings underscore the central role of resources in shaping employee well-being in digitalized work environments. Digital technology support, resilience, and autonomy were each positively associated with job satisfaction and thriving at work (supporting hypotheses H3a–c and H4a–c), reinforcing a core proposition of the JD-R model that resources play a crucial motivational role and are central to explaining how job demands relate to well-being outcomes (36, 47, 99). However, only two mediation pathways were supported: digital innovation support mediated the relationship between digital system overload and job satisfaction, and resilience partially mediated the relationship between digital system overload and thriving at work. One possible explanation is that heightened digital system demands shape how healthcare workers perceive and utilize available digital innovation support, which in turn is associated with their workplace well-being. These results suggest that resources do not function uniformly as mediators but instead vary in salience depending on the nature of the digital demand and the outcome considered.

Third, this study extends JD-R theory by highlighting the importance of demand-specific and context-sensitive resources. Digital technology support, as a resource directly targeting technological complexity, emerged as more theoretically consequential than autonomy, a traditionally central job resource. Despite its positive direct associations with well-being, autonomy did not mediate the relationships of digital job demands. This finding refines JD-R assumptions by suggesting that in highly digitalized healthcare environments, generic job resources may be less effective in buffering digital stressors than resources explicitly aligned with the demands introduced by digital systems.

Finally, the mediating role of psychological resilience further refines theoretical understanding of personal resources within the extended JD-R model. Resilience mediated the relationship between digital system overload and thriving at work but not job satisfaction, indicating that resilience may be particularly relevant for eudaimonic well-being outcomes related to vitality and learning. This supports conceptualizations of resilience as a dynamic, state-like capacity that is activated when employees face challenges threatening adaptability and professional growth, rather than routine workload pressures (126).

Taken together, these findings advance JD-R theory by emphasizing that the well-being implications of digitalization depend less on the presence of digital demands per se and more on the availability and fit of context-specific resources that enable employees to adapt, cope, and grow in digitally intensive work environments.

### 5.2 Practical implications

The findings of this study have four implications for healthcare organizations and managers responsible for digital transformation. First, the results suggest that digital job demands do not uniformly undermine employee well-being. Digital work overload showed no direct negative associations with job satisfaction or thriving at work. For organizations, this can imply that digital pressures should not automatically be treated as harmful but instead evaluated in terms of which specific demands are most disruptive to employees' work experiences. Managers should therefore differentiate between system-related complexity and workload volume when assessing digital strain, rather than assuming a general overload effect.

Second, the findings highlight the importance of organizational-level, demand-specific resources. Digital technology support (DTS) emerged as a key resource, both directly enhancing well-being and mediating the negative effects of digital system overload on job satisfaction. From a managerial perspective, this underscores the need to complement digital system implementation with ongoing technical support, user-oriented training, and accessible troubleshooting mechanisms. Investing in such support structures may reduce system-related frustration and enable healthcare workers to focus more effectively on patient care, consistent with research emphasizing the role of organizational resources in sustaining well-being in demanding work environments.

Third, the mediating role of psychological resilience in the relationship between digital system overload and thriving at work suggests that personal resources remain relevant, particularly for sustaining vitality and learning in complex digital settings. Healthcare organizations may therefore benefit from integrating resilience-building elements into professional development initiatives, such as reflective practices, peer support, and learning-oriented work climates (127). Importantly, these initiatives should complement, not substitute for, organizational responsibility for providing adequate digital support.

Finally, the findings refine the role of autonomy in digitalized healthcare work. Although autonomy was positively associated with well-being outcomes, it did not buffer the effects of digital job demands. This suggests that granting discretion alone may be insufficient when employees face complex digital systems without adequate support. Managers should therefore view autonomy as effective only in combination with demand-specific resources, rather than as a standalone solution to digital strain.

Overall, the results suggest that successful digital transformation in healthcare requires moving beyond a narrow focus on technology adoption or efficiency metrics. By embedding digital support and resource development into digital innovation strategies, healthcare organizations may better support employee well-being. This is an important consideration since empirical studies shown the direct relevance of healthcare workers well-being and patient well-being and quality of care (28).

### 5.3 Limitations and future research

This study has several limitations that need to be acknowledged. First, this study relied on cross-sectional, self-reported data collected via a nonprobability purposive sampling strategy via Prolific. The use of Prolific ensured high-quality participant recruitment (110). Nevertheless, such designs introduce several potential biases. These include self-selection bias from prescreening filters, platform-specific characteristics (e.g., compensation effects), and reduced variability due to targeted participant criteria. Furthermore, the rapidly evolving nature of online platforms can pose challenges to the replicability of data collection procedures over time (110).

Second, the cross-sectional nature of the data constrains the ability to infer causality. While the hypothesized model was grounded in prior empirical research and was tested via structural equation modeling (SEM), this technique does not permit conclusions about temporal ordering (128). As a result, the observed mediating effects should be interpreted as associational rather than causal. Future research would benefit from employing longitudinal or experimental designs to examine how digital demands and resources evolve and how their effects on well-being develop or shift in response to technological changes (128, 129).

Third, the generalizability of the findings is limited because of the specificity of the sample: UK-based health care professionals, with an overrepresentation of women and nurses. Moreover, the use of non-probability sampling through Prolific may introduce selection bias and further limit generalizability. While the focus on health care workers allows for depth in a critical sector, the findings may not be extrapolated to other roles, industries, or national contexts. Variability in digital infrastructure, organizational culture, and work role expectations could shape how digital demands are experienced and managed (130). Future studies should consider comparing findings across sectors and countries to test the robustness of the model. In addition, future research can explore the variations of digital innovations to provide nuanced findings, such as artificial intelligence and its role on the work environment.

Fourth, although precautions were taken to reduce bias (e.g., anonymous data collection and randomized item presentation), common-method variance remains a potential concern due to the use of self-reported measures collected at a single time point. This study followed the recommendations of Kock et al. (131) in testing for common method bias (CMB), however, the exclusive reliance on self-reported data still poses the risk of residual CMB. This design increases the risk of common method variance, which could inflate the relationships between the constructs (132). Future research would benefit from triangulating self-report data with population-level data or from integrating alternative data sources (e.g., observational or administrative data) to reduce bias and increase generalizability. For example, Tsekouropoulos et al. (133) call for more research to explore the evolving challenges that health care professionals face as they navigate ongoing and future digital transformations in the health care sector and beyond.

Accordingly, several areas warrant further investigation to deepen our understanding of the conditions under which digital job demands function as either enablers of or barriers to employee well-being. First, the study can be replicated in other sectors, and comparisons to population data can be conducted to explore possible discrepancies or variations in health care across different nations. Additionally, other resources, such as leadership style and other DJDs, including information overload, can be further investigated. The field would also benefit from exploring underlying mechanisms and how contextual factors, such as job roles, the industry, and organizational culture, shape employees' perceptions of digital demands as either challenges or hindrances (37, 134). Most of the participants in this study are women and nurses, and as a result, future research can further investigate gender and occupational differences as factors in furthering the knowledge of how to navigate digital demands in the health care sector. As noted above, a longitudinal perspective would be especially valuable for testing mediating effects and understanding how resources and demands evolve over time. This is evident, as having more or less of the same resources (135) and job demands can, in the short term, become a challenge. In the long term, resources can also act as a hindrance and negatively impact the well-being of health care workers (36).

Moreover, future research could benefit from adopting complementary theoretical perspectives to extend the present findings. For instance, Conservation of Resources (COR) theory could offer a broader understanding of resource gain and loss cycles as employees adapt to technological change, whereas Self-Determination Theory (SDT) could provide deeper insights into how digital environments support or undermine basic psychological needs such as autonomy, competence, and relatedness. Examining digital innovations through these lenses could enrich understanding of how technological and human factors jointly shape employee well-being and motivation in healthcare. Additionally, rather than viewing employees as passive recipients of digital technologies, our findings suggest that future research can benefit from exploring how employees actively navigate digital work environments, including how they leverage resources, cope with job demands, and exercise agency in response to digitalization.

## 6 Conclusion

This study offers nuanced insights into how digital job demands and key resources are related to employee well-being in health care settings. The findings underscore the complexity of digital system use and its differentiated effects on outcomes like job satisfaction and thriving at work. While digital innovation support, resilience, and autonomy were positively associated with well-being, their mediating roles were limited, as only digital innovation support mediated the effect of digital system overload on job satisfaction, and resilience mediated the relationship between system overload and thriving. The absence of significant effects for digital work overload, and the mixed mediation results, highlight that digital demands may not uniformly function as stressors. Their impact likely is shaped by contextual appraisals and the availability of targeted support. Theoretically, the study contributes to the ongoing development of the Job Demands–Resources model in digitalized work environments, especially within health care. Practically, the findings suggest that generic resources may not always mediate the effects of digital demands. Instead, domain-specific supports like digital innovation support may play a more pivotal role in supporting employee well-being. As health systems continue to adopt digital tools, organizations must consider not only implementation but also the evolving resource needs of their workforce.

Overall, this study contributes to the growing field of digital health services research by emphasizing the importance of identifying which resources remain most relevant and effective in today's digitally transforming health care environments.

## Data Availability

The raw data supporting the conclusions of this article will be made available by the authors, without undue reservation.
