# Supplementary material for: Digital job demands and healthcare workers' workplace well-being: the mediating role of job and personal resources
Source: Front Health Serv. 2026 Feb 27;6:1743364. doi: 10.3389/frhs.2026.1743364 (PMC12982379; doi:10.3389/frhs.2026.1743364)
Supplement: Supplementary file 1 [file Supplementaryfile1.docx]

**Appendix A: Descriptive statistics (n = 292)**

| **Characteristics** | | **N** | **%** |
| --- | --- | --- | --- |
| **Education level** | Bachelor’s Degree | 162 | 55% |
|  | Master’s Degree | 56 | 19% |
|  | Doctoral/Professional Degree | 32 | 11% |
|  | High School Diploma | 28 | 10% |
|  | Associate Degree | 11 | 4% |
|  | Others | 3 | 1% |
| **Employment tenure** | 0-5 years | 146 | 50% |
|  | 6-12 years | 76 | 26% |
|  | 13-20 years | 36 | 12% |
|  | 20+ years | 33 | 11% |
|  | Other | 1 | 0% |
| **Employment type** | Full-time | 191 | 65% |
|  | Part-time | 78 | 27% |
|  | Casual/Seasonal | 6 | 2% |
|  | Freelancer | 6 | 2% |
|  | Temporary/Contract | 4 | 1% |
|  | Self-Employed | 3 | 1% |
|  | Per Diem | 2 | 1% |
|  | Other | 2 | 1% |
| **Gender** | Female | 224 | 77% |
|  | Male | 68 | 23% |
| **Leadership role** | Team Leader | 103 | 35% |
|  | Entry-Level Staff | 79 | 27% |
|  | No Leadership Role | 53 | 18% |
|  | Department Manager | 26 | 9% |
|  | Other | 24 | 8% |
|  | Executive Leadership | 7 | 2% |
| **Occupational role** | Nurse | 191 | 65% |
|  | Physician | 44 | 15% |
|  | Other Clinical | 30 | 10% |
|  | Paramedic | 18 | 6% |
|  | Administration | 9 | 3% |
| **Organization type** | Public Organization | 229 | 78% |
|  | Private Organization | 40 | 14% |
|  | Non-Profit Organization | 14 | 5% |
|  | Academic Medical Center | 7 | 2% |
|  | Other | 2 | 1% |
| **Years of Experience in healthcare** | 6-12 years | 88 | 30% |
|  | 20+ years | 83 | 28% |
|  | 0-5 years | 65 | 22% |
|  | 13-20 years | 52 | 18% |
|  | Others | 4 | 1% |
