# Supplementary material for: Digital job demands and healthcare workers' workplace well-being: the mediating role of job and personal resources
Source: Front Health Serv. 2026 Feb 27;6:1743364. doi: 10.3389/frhs.2026.1743364 (PMC12982379; doi:10.3389/frhs.2026.1743364)
Supplement: Supplementary file 2 [file Supplementaryfile2.docx]

**Appendix B Initial Measurement Model: Item Retention and Deletion During Purification**

|  |  | **Factor loading** | **Mean** | **SD** | | **CR** | **AVE** | | | |
| --- | --- | --- | --- | --- | --- | --- | --- | --- | --- | --- |
| Digital work overload | |  | 4.68 | 1.45 | | 0.89 | 0.73 | | |  |
| demwork1 | | .86 |  |  | |  |  | | |  |
| demwork2 | | .77 |  |  | |  |  | | |  |
| demwork3 | | .91 |  |  | |  |  | | |  |
| Digital system overload | |  | 4.55 | 1.28 | | 0.82 | 0.53 | | |  |
| demsys1 | | .60 |  |  | |  |  | | |  |
| demsys2 | | .81 |  |  | |  |  | | |  |
| demsys3 | | .81 |  |  | |  |  | | |  |
| demsys4 | | .65 |  |  | |  |  | | |  |
| DTS |  |  | 4.56 | 1.27 | | 0.87 | 0.59 | | |  |
| icts1 | | .84 |  |  | |  |  | | |  |
| icts2 | | .85 |  |  | |  |  | | |  |
| icts3 | | .87 |  |  | |  |  | | |  |
| icts4 | | .66 |  |  | |  |  | | |  |
| icts5 | | .61 |  |  | |  |  | | |  |
| Psychological resilience | | | 4.25 | 1.28 | | 0.93 | 0.72 | | |  |
| res1 | | .78 |  |  | |  |  | | |  |
| res2 | | .78 |  |  | |  |  | | |  |
| res3 | | .87 |  |  | |  |  | | |  |
| res4 | | .88 |  |  | |  |  | | |  |
| res5 | | .85 |  |  | |  |  | | |  |
| res6 | | .91 |  |  | |  |  | | |  |
| TAW |  |  | 4.75 | 0.63 | | 0.75 | 0.53 | | |  |
| TAW1* | | .559 |  |  | |  |  | | |  |
| TAW2* | | .575 |  |  | |  |  | | |  |
| TAW3 | | .72 |  |  | |  |  | | |  |
| TAW4* | | -.424 |  |  | |  |  | | |  |
| TAW5 | | .73 |  |  | |  |  | | |  |
| TAW6 | | .88 |  |  | |  |  | | |  |
| TAW7 | | .89 |  |  | |  |  | | |  |
| TAW8 | | .77 |  |  | |  |  | | |  |
| JS |  |  | 4.75 | 1.32 | | 0.93 | 0.76 | | |  |
| job1 | | .84. |  |  | |  |  | | |  |
| job2 | | .91 |  |  | |  |  | | |  |
| job3 | | .79 |  |  | |  |  | | |  |
| job4 | | .94 |  |  | |  |  | | |  |
| Autonomy |  |  | 5.1 | 1.18 | | 0.76 | 0.502 | | |  |
| aut1* | | .487 |  |  |  | |  | | | |
| aut2* | | .530 |  |  |  | |  | | | |
| aut3 | | .75 |  |  |  | |  | | | |
| aut4 | | .92 |  |  |  | |  | | | |
| aut5 | | .91 |  | | | |  |  |  |  |

Note: SD: Standard deviation; CR: Composite reliability; AVE: Average variance extracted; DWO: digital work overload; DSO: digital system overload; DTS: digital technology support; Resilience: psychological resilience; TAW: Thriving at work; JS: job satisfaction; *Items dropped during the purification process due to low standardized factor loadings (<0.6). n = 292
